# Single‐Cell and Bulk Transcriptomic Integration Reveals an Adverse SPP1 Inflammatory TAM State and INHBA as a Candidate Biomarker in Non‐Small Cell Lung Cancer Immunotherapy

**DOI:** 10.1155/ijog/2321522

**Published:** 2026-08-26

**Authors:** Sheng Zhang, Guojun Zhang, Wenxuan Wang, Hong Luo, Huaping Chen

**Affiliations:** ^1^ Department of Radiology, Beijing Anzhen Nanchong Hospital of Medical University & Nanchong Central Hospital, Nanchong, China; ^2^ Department of Oncology, Beijing Anzhen Nanchong Hospital of Medical University & Nanchong Central Hospital, Nanchong, China

**Keywords:** biomarker discovery, bulk RNA-seq, INHBA, neoadjuvant immunotherapy, non-small cell lung cancer, single-cell RNA sequencing, SPP1 inflammatory TAM, tumor-associated macrophages

## Abstract

**Background:**

Biomarkers that capture the microenvironmental basis of response to neoadjuvant immunotherapy in non‐small cell lung cancer (NSCLC) remain limited. We integrated single‐cell and bulk transcriptomic data to identify response‐associated immune programs with patient‐level relevance.

**Methods:**

Single‐cell RNA‐seq and clinical annotations from GSE207422 were used to construct an NSCLC tumor microenvironment atlas and refine myeloid and T cell states. UCell scoring, sample‐level summaries, pseudobulk differential analysis, Monocle2 trajectory inference, and targeted macrophage–T cell interaction analysis were applied. A posttreatment SPP1 inflammatory TAM‐derived NR‐TAM signature was projected to pretreatment bulk RNA‐seq from the same cohort. INHBA was evaluated in pretreatment bulk data, TCGA‐LUAD/TCGA‐LUSC, and lung cancer cell line assays.

**Results:**

SPP1 inflammatory TAM was enriched in nonmajor pathologic response (NMPR) samples and co‐occurred with exhausted CD8 T cell abundance, malignant cell hypoxia, and EMT programs. The NR‐TAM signature was partially recapitulated in pretreatment bulk RNA‐seq, with higher scores in NMPR but substantial group overlap. Exploratory ROC analysis showed modest discrimination for NR‐TAM score (AUC = 0.60), INHBA (AUC = 0.64), and the combined model (AUC = 0.64; LOOCV AUC = 0.37). INHBA was higher in NMPR cases, correlated with residual tumor burden and selected TAM‐related genes, showed less favorable survival trends particularly in LUSC, and its knockdown reduced proliferation and migration in lung cancer cells.

**Conclusions:**

An SPP1 inflammatory TAM program is associated with unfavorable pathologic response in NSCLC neoadjuvant immunotherapy. INHBA is a prioritized exploratory candidate within this program. Because the evidence is associative and predictive performance is modest, these markers require prospective validation before clinical use.

## 1. Introduction

Non‐small cell lung cancer (NSCLC) remains the leading cause of cancer‐related mortality worldwide and continues to impose a substantial clinical burden despite advances in surgery, systemic therapy, and biomarker‐guided management [1, 2]. In recent years, immune checkpoint blockade, particularly in the neoadjuvant setting, has reshaped the therapeutic landscape of resectable NSCLC by improving pathologic response and expanding opportunities for earlier systemic intervention [3–6]. However, the clinical benefit of immunotherapy is heterogeneous, and a considerable proportion of patients fail to achieve major pathologic response or durable disease control [7–9]. This variability underscores the need to identify biomarkers that more precisely capture the biological states associated with treatment sensitivity and resistance.

Current efforts to predict immunotherapy response have largely relied on bulk‐level features, including PD‐L1 expression, tumor mutational burden, and immune‐related transcriptional signatures [10–13]. Although these markers provide useful clinical information, their performance remains incomplete, in part because bulk measurements average signals across malignant, immune, and stromal compartments and therefore obscure the cellular architecture of the tumor microenvironment (TME) [14, 15]. This limitation is particularly relevant in NSCLC, where treatment response is strongly shaped not only by tumor‐intrinsic properties but also by the composition, functional state, and spatial organization of immune and stromal cell populations [16, 17]. As a result, biomarkers defined without cellular resolution may fail to capture the specific microenvironmental programs that accompany unfavorable therapeutic outcomes.

Single‐cell RNA sequencing (scRNA‐seq) provides an opportunity to overcome this limitation by resolving the cellular heterogeneity of the TME and identifying lineage‐specific programs that are masked in bulk analyses [18, 19]. In NSCLC, single‐cell studies have revealed substantial diversity among lymphoid, myeloid, and epithelial compartments and have shown that immunotherapy response is associated with coordinated remodeling of multiple cell states rather than a single immune variable [20–22]. Among these components, tumor‐associated macrophages (TAMs) are of particular interest because they occupy a central position at the interface of inflammation, stromal remodeling, antigen presentation, immune suppression, and therapeutic response [23, 24]. Rather than constituting a uniform population, TAMs comprise multiple transcriptionally and functionally distinct states, some of which may support antitumor immunity, whereas others may reinforce immune dysfunction and tumor progression [25, 26]. Identifying the specific TAM states linked to poor response may therefore yield more informative biomarkers than bulk macrophage abundance alone.

At the same time, single‐cell discoveries face an important translational challenge. Although scRNA‐seq can define high‐resolution cell states, most clinically annotated cohorts and validation datasets remain bulk transcriptomic. For this reason, an effective biomarker discovery strategy should not stop at identifying a cell state at single‐cell resolution but should also determine whether its transcriptional features can be projected to patient‐level bulk data in a reproducible and clinically meaningful manner [27, 28]. Integrating single‐cell and bulk transcriptomic analyses may therefore offer a practical route to bridge biological resolution and clinical applicability.

In the present study, we used an integrative single‐cell and bulk transcriptomic framework to identify immune microenvironmental states associated with pathologic response to neoadjuvant immunotherapy in NSCLC. By constructing a TME atlas from GSE207422 and refining the myeloid and T cell compartments, we identified a distinct SPP1 inflammatory TAM state enriched in samples with nonmajor pathologic response (NMPR). This macrophage state was accompanied by features of an unfavorable immune ecosystem, including increased exhausted CD8 T cell abundance and higher malignant cell hypoxia and epithelial–mesenchymal transition programs. We further derived an adverse response–associated NR‐TAM signature from pseudobulk analysis of this TAM population and projected it to pretreatment bulk RNA‐seq data from the same cohort. Among the prioritized candidate genes, INHBA emerged as a representative molecule linked to poor pathologic response, residual tumor burden, and unfavorable survival trends in external lung cancer cohorts. Finally, functional experiments in lung cancer cell lines supported the tumor‐associated expression and phenotypic relevance of INHBA. Together, this study provides an integrative framework for identifying clinically relevant immune programs in NSCLC immunotherapy and highlights an adverse response–associated macrophage axis with potential biomarker value.

## 2. Materials and Methods

### 2.1. Data Sources and Study Design

This study integrated publicly available single‐cell and bulk transcriptomic datasets to characterize tumor microenvironmental states associated with pathologic response to neoadjuvant immunotherapy in NSCLC. Single‐cell RNA‐seq data and matched clinical annotations were obtained from GSE207422 and used to construct the cellular atlas and perform cell state analyses [20, 29]. According to the available annotations, 15 immune‐related samples were included in the single‐cell analysis, comprising 12 posttreatment surgical specimens and three pretreatment biopsy specimens. In parallel, bulk RNA‐seq data from the same cohort were used to evaluate whether single cell–derived transcriptional features could be projected to the patient level and to assess the clinical relevance of candidate genes [30]. A total of 24 pretreatment bulk RNA‐seq samples were available for analysis, including 15 NMPR cases and 9 major pathologic response/pathologic complete response (MPR/pCR) cases. In addition, transcriptomic and clinical data from TCGA‐LUAD and TCGA‐LUSC were analyzed as external reference cohorts for expression and survival analyses of selected candidate genes [31, 32]. Pathologic response groups were harmonized according to the original annotations, and for binary comparisons, MPR and pCR were combined as the MPR/pCR group.

### 2.2. Single‐Cell RNA‐Seq Preprocessing and Quality Control

The single‐cell expression matrix and sample annotation table were matched by sample identifiers and imported into Seurat for downstream analysis [33]. Quality control was performed based on the number of detected genes per cell (nFeature_RNA), the percentage of mitochondrial transcripts (percent.mt), and the percentage of hemoglobin‐related transcripts (percent.HB) [34]. Cells with low transcript complexity or elevated mitochondrial/hemoglobin signals were considered likely to represent low‐quality cells, stressed cells, or contaminated droplets and were removed before downstream analyses. The distributions of these quality metrics before and after filtering were visualized using violin plots stratified by patient to illustrate the effect of filtering on overall data quality.

### 2.3. Dimensionality Reduction, Integration, Clustering, and Major Cell–Type Annotation

After quality control, single cell data were processed using the standard Seurat workflow, including normalization, identification of highly variable genes, data scaling, and principal component analysis. To reduce intersample batch effects, Harmony was applied using sample identity as the integration variable [35]. The integrated low‐dimensional embeddings were then used for UMAP visualization and graph‐based clustering. Major cellular compartments were annotated according to cluster‐specific marker genes and canonical lineage markers. Based on this procedure, the main cell populations were defined as T cells, B cells, plasma cells, myeloid cells, neutrophils, mast cells, fibroblasts, AT2 epithelial cells, club cells, malignant epithelial cells, and cycling cells.

### 2.4. Refinement of Myeloid and T Cell Subsets

To further resolve intracompartmental heterogeneity within the immune microenvironment, myeloid cells and T cells were extracted separately and reanalyzed. For each compartment, normalization, dimensionality reduction, integration, and clustering were repeated using the same general framework as described above. Subclusters were manually annotated according to their marker expression patterns. Myeloid cells were subdivided into monocyte, resident macrophage, IFN response macrophage, APOC1_GPNMB TAM, SPP1 inflammatory TAM, and dendritic‐cell subsets including cDC2, pDC, and mregDC [36]. T cells were further resolved into Exhausted CD8 T, Effector CD8 T, naive/memory T, Treg, CXCL13+ CD4 T, and NK cells [37, 38].

### 2.5. Cell State Scoring and Sample‐Level Summarization

To quantify biologically relevant transcriptional programs at single‐cell resolution, UCell was applied to calculate signature scores for selected cell states [39]. The evaluated programs included TAM‐related states, T cell exhaustion, cytotoxicity, Treg‐associated programs, and malignant cell phenotypes such as hypoxia, epithelial–mesenchymal transition (EMT), and interferon response. Based on refined cell annotations and single‐cell state scores, sample‐level summaries were generated by calculating the proportions of key cell states and the average malignant cell state scores within each sample. These metrics were subsequently used for group comparison and correlative analyses across pathologic response categories.

### 2.6. Differential Characterization of SPP1 Inflammatory TAM and Derivation of the NR‐TAM Signature

Within the myeloid compartment, SPP1 inflammatory TAM was compared with the remaining myeloid subsets to identify its representative marker genes [40]. To reduce the influence of pseudoreplication inherent to cell‐level testing, pseudobulk expression profiles were generated for posttreatment samples by aggregating raw counts at the sample‐by‐cell‐subset level. Differential expression between NMPR and MPR/pCR groups within the SPP1 inflammatory TAM compartment was then assessed using the edgeR and limma‐voom framework [41–43]. Genes showing stronger upregulation trends in the NMPR group were prioritized as candidate genes and were used to derive a composite NR‐TAM signature representing the adverse response–associated transcriptional pattern of SPP1 inflammatory TAM.

### 2.7. Trajectory Inference of Monocyte‐To‐TAM Transition

To investigate continuous transitions among myeloid states, monocyte, APOC1_GPNMB TAM, and SPP1 inflammatory TAM were selected for trajectory analysis using Monocle2. After moderate down‐sampling of overly abundant populations, a trajectory object was constructed from raw‐count data and pseudotime was inferred [44]. Genes dynamically changing along pseudotime were identified to characterize the transcriptional progression from monocyte‐like states toward terminal TAM programs.

### 2.8. Evaluation of Macrophage–T Cell Interactions

To examine immune interaction patterns relevant to treatment response, we focused on selected macrophage–T cell ligand–receptor axes rather than reconstructing a full communication network. The analysis was restricted to major myeloid sender populations and key T cell receiver populations in posttreatment samples. For each sample and cell type, average gene expression was calculated, and interaction strength was defined according to the joint expression of ligands in sender cells and their corresponding receptors in receiver cells. This strategy enabled sample‐level comparison of targeted interaction landscapes between response groups while maintaining interpretability for predefined immune axes.

### 2.9. Projection of the NR‐TAM Signature to Pretreatment Bulk RNA‐Seq

For the pretreatment bulk RNA‐seq data from GSE207422, the expression matrix was organized by gene symbol and duplicate gene entries were collapsed prior to downstream analysis. The NR‐TAM score was then calculated from standardized expression values of the NR‐TAM signature genes to quantify the extent to which the adverse SPP1 inflammatory TAM‐associated transcriptional pattern could be captured at the patient level. The resulting score was used for comparison between pathologic response groups and for visualization of interpatient heterogeneity.

Because the NR‐TAM signature was derived from posttreatment single‐cell profiles and then evaluated in pretreatment bulk RNA‐seq, this projection was interpreted under a defined biological assumption: At least part of the adverse SPP1 inflammatory TAM‐associated transcriptional program may reflect a recurrent tumor‐microenvironmental tendency that can be detected at the bulk level before treatment. This analysis cannot determine whether the program is preexisting, treatment‐induced, or a mixture of both, and bulk RNA‐seq cannot resolve the cellular origin of each transcript. Therefore, the pretreatment bulk analysis was used to test partial patient‐level recapitulation of the single cell–derived program rather than to establish its temporal origin.

### 2.10. Candidate Gene Analysis of INHBA and External TCGA Validation

INHBA was selected as the representative candidate gene for patient‐level evaluation. In the pretreatment bulk RNA‐seq cohort, its expression was compared across pathologic response groups, RECIST categories, and clinical subgroups, and its associations with residual tumor proportion, TAM‐related genes, and the NR‐TAM score were assessed. To further evaluate its broader clinical relevance, transcriptomic and clinical data from TCGA‐LUAD and TCGA‐LUSC were analyzed. Kaplan–Meier survival analysis and Cox proportional hazards regression were performed to examine the prognostic association of INHBA expression in these independent cohorts.

### 2.11. Cell Lines and Cell Culture

The human bronchial epithelial cell line BEAS‐2B (RRID: CVCL_0168), human NSCLC cell lines A549 (RRID: CVCL_0023) and H1299 (RRID: CVCL_0060), and the human embryonic kidney cell line HEK293T (RRID: CVCL_0063) were used in this study. BEAS‐2B cells were obtained from the American Type Culture Collection (ATCC), and A549, H1299, and HEK293T cells were purchased from the Cell Bank of the Chinese Academy of Sciences (Shanghai, China). BEAS‐2B cells were cultured in DMEM/F12 medium (Gibco, Cat#11320033), whereas A549, H1299, and HEK293T cells were maintained in high‐glucose DMEM (Gibco, Cat#11965092). All media were supplemented with 10% fetal bovine serum (FBS; Gibco, Cat#10099141C) and 1% penicillin–streptomycin (Gibco, Cat#15140122). Cells were cultured at 37°C in a humidified incubator with 5% CO_2_ and routinely tested for mycoplasma contamination. Cells within 10 passages after thawing were used for all experiments.

Short hairpin RNAs (shRNAs) targeting INHBA (shINHBA‐1, shINHBA‐2, and shINHBA‐3) and a nontargeting control (shNC) were cloned into the lentiviral vector pLKO.1‐puro (Addgene). Lentiviral particles were produced by cotransfecting HEK293T cells with the shRNA plasmids, psPAX2 packaging plasmid (Addgene, Cat#12260), and pMD2.G envelope plasmid (Addgene, Cat#12259) using Lipofectamine 3000 transfection reagent (Invitrogen, Cat#L3000015). After 48–72 h, viral supernatants were collected, filtered through a 0.45‐*μ*m membrane, and used to infect A549 and H1299 cells in the presence of Polybrene (Sigma‐Aldrich, Cat#TR‐1003‐G). Forty‐eight hours postinfection, cells were selected with puromycin (Sigma‐Aldrich) for 1–2 weeks to establish stable cell lines. Knockdown efficiency was validated by quantitative real‐time PCR (qPCR).

### 2.12. qPCR

Total RNA was extracted from cells using TRIzol reagent (Invitrogen, Cat#15596026) according to the manufacturer′s instructions. RNA concentration and purity were measured using a NanoDrop 2000 spectrophotometer (Thermo Scientific). A total of 1 *μ*g RNA was reverse‐transcribed into cDNA using a reverse transcription kit (Takara, Cat#RR047A). qPCR was performed using SYBR Green Master Mix (Applied Biosystems, Cat#A25742) on a QuantStudio 5 Real‐Time PCR System (Applied Biosystems). The reaction mixture (20 *μ*L) consisted of 10‐*μ*L SYBR Green Master Mix, 0.4‐*μ*L forward primer (10 *μ*M), 0.4‐*μ*L reverse primer (10 *μ*M), 2‐*μ*L cDNA template, and 7.2‐*μ*L RNase‐free water. The amplification conditions were as follows: initial denaturation at 95°C for 10 min, followed by 40 cycles of 95°C for 15 s and 60°C for 60 s. GAPDH was used as an internal control, and relative gene expression levels were calculated using the 2^−*ΔΔ*Ct^ method. BEAS‐2B cells or shNC‐treated cells were used as reference controls as indicated. All experiments were performed in triplicate. The primer sequences used in this study were as follows:

GAPDH‐F: GACAGTCAGCCGCATCTTCT,

GAPDH‐R: GCGCCCAATACGACCAAATC,

INHBA‐F: AGAGGAGCTCAGACAGCTCTTA,

INHBA‐R: TCTCTTTCTGGTCCCCACTCT.

### 2.13. CCK‐8 Cell Proliferation Assay

Cell proliferation was assessed using a Cell Counting Kit‐8 (CCK‐8; Dojindo, Cat#CK04). A549 and H1299 cells transfected with shNC or INHBA‐targeting shRNAs were seeded into 96‐well plates at a density of approximately 2 × 10^3^ cells per well in triplicate. Cells were cultured at 37°C in a humidified incubator with 5% CO_2_. At 0, 24, 48, 72, and 96 h, 10 *μ*L of CCK‐8 reagent was added to each well and incubated for 1–2 h. Absorbance was measured at 450 nm using a microplate reader. The OD values were normalized to the 0‐h time point to generate cell growth curves. All experiments were performed independently at least three times.

### 2.14. Wound Healing Assay

Cell migration was evaluated using a wound healing assay. A549 and H1299 cells transfected with shNC or INHBA‐targeting shRNAs were seeded into six‐well plates and cultured to approximately 90% confluence. A linear scratch was created in the cell monolayer using a sterile 200‐*μ*L pipette tip. Cells were gently washed twice with PBS to remove detached cells and then cultured in medium containing 1% FBS to minimize the effect of cell proliferation. Images of the wound area were captured at 0 and 24 h using an inverted microscope. Wound width was measured using ImageJ software, and the migration rate was calculated as follows: migration rate (%) = (wound width at 0 h − wound width at 24 h)/wound width at 0 h × 100%. Each experiment was performed in triplicate and repeated at least three times.

### 2.15. Statistical Analysis

Single‐cell differential expression analysis was primarily performed using the Wilcoxon rank‐sum test, whereas pseudobulk differential analysis was conducted using the limma‐voom pipeline. Group comparisons in bulk RNA‐seq and TCGA datasets were mainly based on nonparametric tests. Correlation analyses were performed using Spearman′s rank correlation coefficient. Survival analyses, including log‐rank tests and Cox proportional hazards regression, were implemented using the survival package. For cell‐based experiments, comparisons among multiple groups were performed using one‐way analysis of variance (ANOVA) followed by Tukey′s post hoc test. Data from in vitro assays are presented as mean ± standard deviation (SD). Statistical analyses and visualization were performed using R and GraphPad Prism where appropriate. Unless otherwise specified, the statistical results shown in the figures were taken as the primary reporting results, and a two‐sided *p* < 0.05 was considered statistically significant.

To address the potential clinical utility of the NR‐TAM score and INHBA expression, we performed an exploratory predictive performance analysis in the pretreatment GSE207422 bulk RNA‐seq cohort. The NR‐TAM score was recalculated as the mean gene‐wise *Z*‐score of the 12 NR‐TAM signature genes detected in bulk RNA‐seq (SPP1, SIGLEC12, RNASE1, PTGS2, OSM, MT1H, MT1G, MT1E, IL1R2, CXCL1, CCL8, and CCL7). NMPR was coded as the positive class and MPR/pCR as the reference class. Receiver operating characteristic analysis was used to estimate the apparent area under the curve for the NR‐TAM score, INHBA expression, and a two‐variable logistic regression model combining NR‐TAM score and INHBA expression. Because of the small sample size, the combined logistic model was additionally evaluated using leave‐one‐out cross‐validation. Bootstrap resampling was used to estimate exploratory 95% confidence intervals for apparent AUCs. These analyses were considered descriptive and hypothesis‐generating.

## 3. Results

### 3.1. Single‐Cell Atlas Construction and Annotation of Major Cell Populations

We first performed quality control on the GSE207422 single‐cell RNA‐seq data. As shown in Figure 1A, before filtering, multiple samples contained cells with low numbers of detected genes and relatively high mitochondrial transcript fractions. A small proportion of cells also showed detectable hemoglobin‐related transcripts. After filtering, these low‐quality populations were markedly reduced, and the overall distributions of nFeature_RNA, percent.mt, and percent.HB became more restricted across samples, indicating improved data quality. After normalization, integration, and clustering, UMAP analysis identified several major cell populations in the NSCLC microenvironment, including T cells, B cells, plasma cells, myeloid cells, neutrophils, mast cells, fibroblasts, AT2 epithelial cells, club cells, malignant epithelial cells, and cycling cells (Figure 1B). Among them, T cells and myeloid cells represented the major immune compartments, whereas malignant epithelial cells formed a distinct epithelial‐associated cluster. When the global atlas was colored by sample, pathology, specimen source, and RECIST category, the same major cell populations remained readily identifiable across different clinical groupings (Figure 1C). Cells from different samples were distributed within shared cluster structures rather than forming completely separate sample‐specific partitions, suggesting that the integrated atlas preserved common cellular identities across the cohort. The assigned annotations were supported by canonical marker expression patterns (Figure 1D,F). CD3D was mainly expressed in the T cell cluster, MS4A1 in B cells, JCHAIN in plasma cells, LST1 in myeloid cells, S100A8 in neutrophils, COL1A1 in fibroblasts, EPCAM in epithelial compartments, and MKI67 in cycling cells. The dot plot showed the same overall pattern and further supported the reliability of the major cell–type annotation. We next summarized cell composition across specimen sources and pathologic response groups. In both pretreatment biopsy and posttreatment surgery samples, T cells and myeloid cells accounted for a large proportion of the atlas, although the relative fractions of other cell populations varied between groups (Figure 1E). A similar pattern was observed after stratification by pathologic response, where differences were mainly reflected by changes in relative cell proportions rather than the appearance of new major cell classes (Figure 1G). Together, these results established the single‐cell landscape used for subsequent analyses of myeloid and T cell heterogeneity.

Figure 1Construction of the GSE207422 single‐cell atlas and annotation of major cell populations. (A) Violin plots showing quality‐control metrics before and after filtering across samples, including nFeature_RNA, percent.mt, and percent.HB. Red dashed lines indicate the filtering thresholds used in the analysis. (B) UMAP of the integrated single‐cell atlas colored by major cell–type annotations, including T cells, B cells, plasma cells, myeloid cells, neutrophils, mast cells, fibroblasts, AT2 epithelial cells, club cells, malignant epithelial cells, and cycling cells. (C) UMAPs colored by sample identity, pathology, specimen source, and RECIST category. (D) Feature plots showing representative canonical markers, including CD3D, MS4A1, JCHAIN, LST1, S100A8, COL1A1, EPCAM, and MKI67. (E) Stacked bar plot showing cell‐type composition by specimen source. (F) Dot plot showing canonical marker expression across major cell types. Dot size indicates the fraction of expressing cells, and color indicates the average expression level. (G) Stacked bar plot showing cell‐type composition across pathologic response groups.

**Figure:**
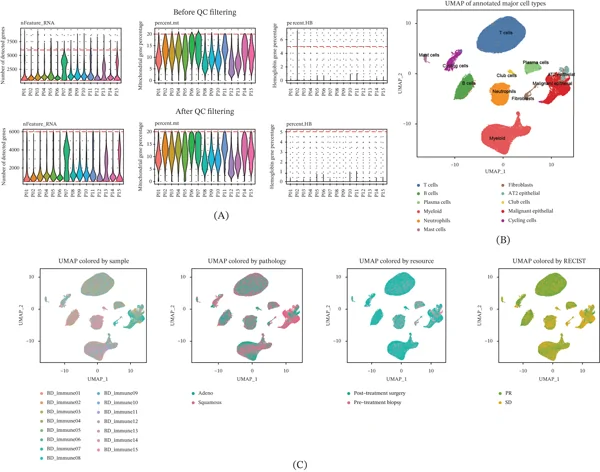
(A)

**Figure:**
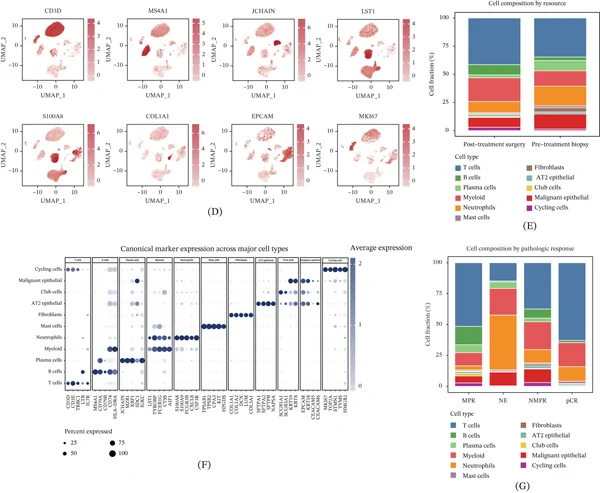
(B)

### 3.2. Refined Annotation of Myeloid and T Cell Subsets and Identification of Key Cellular States

To further resolve immune heterogeneity, we separately re‐clustered myeloid cells and T cells. The myeloid compartment was refined into APOC1_GPNMB TAM, monocyte, resident macrophage, IFN response macrophage, SPP1 inflammatory TAM, cDC2, pDC, and mregDC (Figure 2A). The corresponding marker dot plot supported these annotations, with APOC1/GPNMB enriched in APOC1_GPNMB TAM, SPP1 enriched in SPP1 inflammatory TAM, and FCN1/VCAN enriched in monocytes (Figure 2B). T cell reclustering identified exhausted CD8 T, effector CD8 T, naive/memory T, Treg, CXCL13+ CD4 T, and NK cells (Figure 2C). Marker expression patterns were consistent with these annotations, including exhaustion‐associated markers in exhausted CD8 T cells, cytotoxic markers in effector CD8 T cells, IL7R/CCR7/TCF7 in naive/memory T cells, FOXP3/IL2RA/CTLA4 in Treg cells, and FCGR3A/KLRD1/NCR1 in NK cells (Figure 2D). After transferring the refined annotations back to the full atlas, myeloid and T cell subpopulations remained clearly localized within their corresponding major compartments (Figure 2E). UCell scoring further showed that the SPP1_TAM score was highest in SPP1 inflammatory TAM, the APOC1_GPNMB_TAM score was highest in APOC1_GPNMB TAM, and the T_exhaustion score was most prominent in exhausted CD8 T cells. In contrast, hypoxia and EMT scores were mainly concentrated in malignant epithelial cells, whereas the IFN_response score was more evident in myeloid populations (Figure 2F,G). At the sample level, the fraction of SPP1 inflammatory TAM was higher in the NMPR group than in the MPR/pCR group (Figure 2H). In addition, samples with a higher SPP1 inflammatory TAM fraction tended to show a higher proportion of exhausted CD8 T cells, a higher mean hypoxia score in malignant cells, and a higher mean EMT score in malignant cells (Figure 2I). These results indicate that SPP1 inflammatory TAM is associated with an unfavorable immune and malignant cell state.

Figure 2Refined annotation of myeloid and T cell subsets and overview of key cellular states. (A) UMAP of reclustered myeloid cells showing annotated myeloid subsets, including APOC1_GPNMB TAM, monocyte, resident macrophage, IFN response macrophage, SPP1 inflammatory TAM, cDC2, pDC, and mregDC. (B) Dot plot showing canonical marker expression across myeloid subsets. Dot size indicates the fraction of expressing cells, and color indicates the average expression level. (C) UMAP of reclustered T cells showing annotated T cell subsets, including exhausted CD8 T, effector CD8 T, naive/memory T, Treg, CXCL13+ CD4 T, and NK cells. (D) Dot plot showing canonical marker expression across T cell subsets. (E) UMAP of the full atlas after reintegration of refined myeloid and T cell annotations. (F) Feature plots showing UCell scores for selected signatures, including SPP1_TAM, APOC1_GPNMB_TAM, T_exhaustion, hypoxia, EMT, and IFN_response. (G) Violin plots showing UCell score distributions across key refined cell states. (H) Boxplots showing sample‐level fractions of selected cell states in posttreatment samples stratified by pathologic response. (I) Scatter plots showing the relationships of the SPP1 inflammatory TAM fraction with the exhausted CD8 T cell fraction, mean hypoxia score in malignant cells, and mean EMT score in malignant cells. Each point represents one sample, and black lines indicate linear trends.

**Figure:**
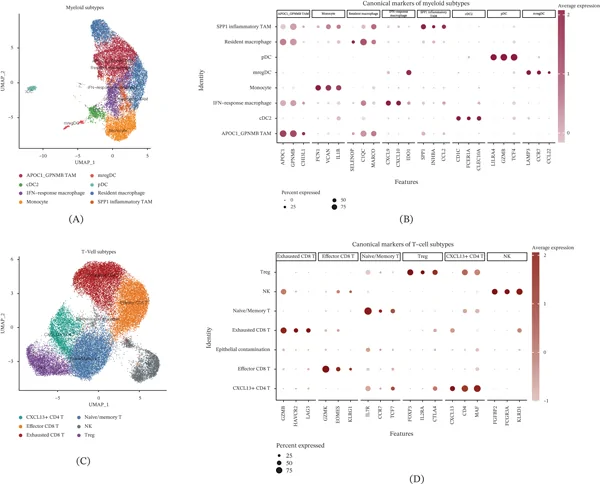
(A)

**Figure:**
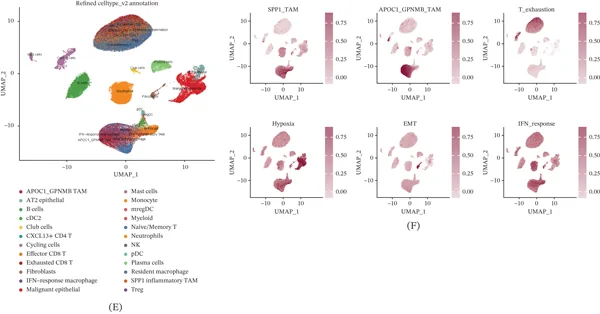
(B)

**Figure:**
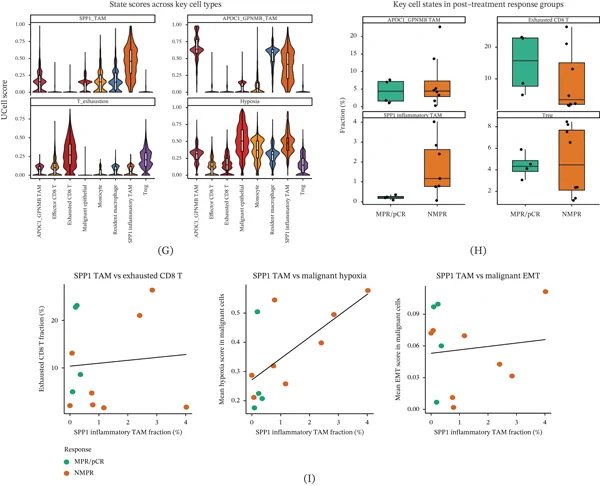
(C)

### 3.3. Molecular Characterization of SPP1 Inflammatory TAM and Derivation of the NR‐TAM Signature

We next focused on SPP1 inflammatory TAM. In the full atlas, this population was localized within the myeloid compartment (Figure 3A). In the myeloid UMAP, the SPP1_TAM score was most enriched in this subset, whereas the APOC1_GPNMB_TAM and IFN_Macrophage scores showed distinct distribution patterns, further distinguishing SPP1 inflammatory TAM from other macrophage states (Figure 3B). At the sample level, SPP1 inflammatory TAM showed a higher fraction in the NMPR group than in the MPR/pCR group, whether calculated across all cells or within the myeloid compartment (Figure 3C). Feature plots showed that this population expressed a characteristic inflammatory program, including SPP1, INHBA, CCL2, CCL7, CXCL8, ADM, APOC1, and GPNMB (Figure 3D). The dot plot further supported its transcriptional distinction from other myeloid subsets (Figure 3E). Consistent with the sample‐level trends observed above, a higher fraction of SPP1 inflammatory TAM was accompanied by a higher proportion of exhausted CD8 T cells, a higher mean hypoxia score in malignant cells, and a higher mean EMT score in malignant cells (Figure 3F). We then performed pseudobulk analysis of SPP1 inflammatory TAM in posttreatment samples. PCA showed transcriptional heterogeneity across samples, with partial separation between response groups (Figure 3G). The heat map of top NMPR‐up genes showed a coordinated expression pattern across samples (Figure 3H), and several representative genes, including ADM, CCL2, CCL7, CXCL8, INHBA, and SPP1, showed higher expression trends in the NMPR group (Figure 3I). Based on these candidate genes, we derived an NR‐TAM signature, whose score was overall higher in NMPR than in MPR/pCR within the SPP1 inflammatory TAM compartment (Figure 3J). Together, these results identify SPP1 inflammatory TAM as a distinct adverse response–associated macrophage state.

Figure 3Molecular characterization of SPP1 inflammatory TAM and derivation of the NR‐TAM signature. (A) UMAP of the full atlas highlighting SPP1 inflammatory TAM cells. (B) Feature plots showing UCell scores for SPP1_TAM, APOC1_GPNMB_TAM, and IFN_Macrophage within the myeloid compartment. (C) Boxplots comparing the fraction of SPP1 inflammatory TAM between MPR/pCR and NMPR groups, calculated across all cells and within the myeloid compartment. (D) Feature plots showing representative genes enriched in SPP1 inflammatory TAM, including SPP1, INHBA, CCL2, CCL7, CXCL8, ADM, APOC1, and GPNMB. (E) Dot plot showing top marker genes of SPP1 inflammatory TAM relative to other myeloid subsets. Dot size indicates the fraction of expressing cells, and color indicates the average expression level. (F) Scatterplots showing the relationships of the SPP1 inflammatory TAM fraction with the exhausted CD8 T cell fraction, mean hypoxia score in malignant cells, and mean EMT score in malignant cells. (G) Principal component analysis of pseudobulk expression profiles of SPP1 inflammatory TAM across posttreatment samples. (H) Heatmap showing genes with stronger upregulation trends in NMPR in the SPP1 inflammatory TAM pseudobulk analysis. Colors represent gene‐wise *Z*‐scores. (I) Boxplots showing representative candidate genes in the SPP1 inflammatory TAM pseudobulk analysis stratified by pathologic response. (J) Boxplot showing the NR‐TAM signature score in SPP1 inflammatory TAM stratified by pathologic response.

**Figure:**
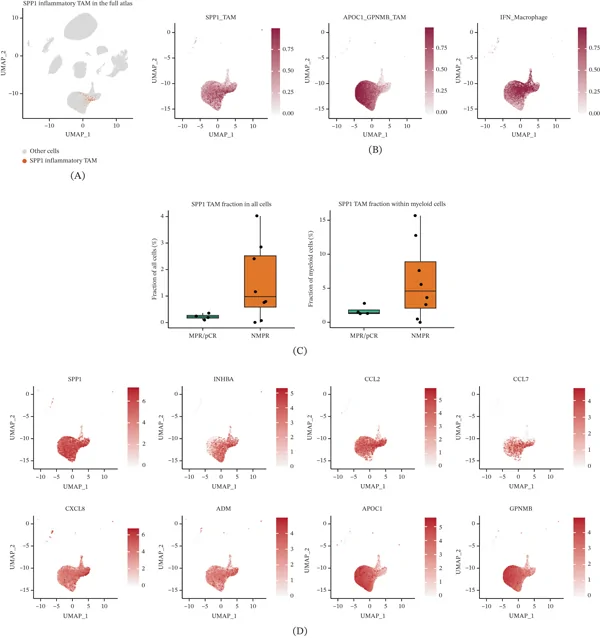
(A)

**Figure:**
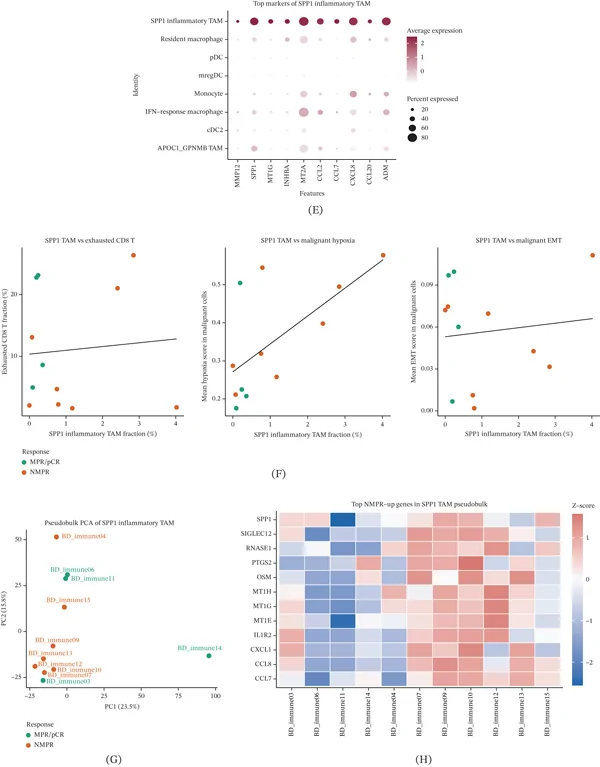
(B)

**Figure:**
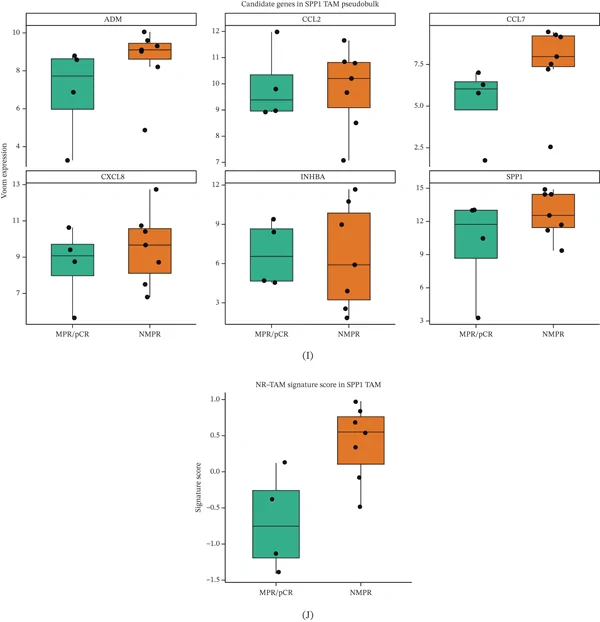
(C)

### 3.4. Monocyte‐To‐TAM Trajectory and SPP1‐Associated Macrophage–T Cell Interaction Landscape

To examine transcriptional ordering among major myeloid states, we performed trajectory analysis using monocyte, APOC1_GPNMB TAM, and SPP1 inflammatory TAM. The trajectory structure placed monocyte‐like cells toward one end of the inferred trajectory, whereas APOC1_GPNMB TAM and SPP1 inflammatory TAM occupied more distal regions (Figure 4A–C). Dynamic expression patterns were compatible with this ordering: FCN1 and VCAN were higher in monocyte‐like regions, whereas APOC1, GPNMB, CCL2, INHBA, and SPP1 increased toward TAM‐enriched regions (Figure 4D). We interpret these results as a computational transcriptional continuum rather than direct evidence of lineage conversion, because trajectory inference cannot exclude alternative differentiation routes or treatment‐related remodeling. We next evaluated macrophage–T cell interaction patterns at the sample level. The overall interaction heat map showed that SPP1 inflammatory TAM had higher inferred interaction scores with multiple T cell subsets, including CXCL13+ CD4 T, effector CD8 T, exhausted CD8 T, and Treg cells (Figure 4E). A focused view of top interaction pairs highlighted SPP1‐related ligand–receptor axes, particularly SPP1‐ITGB1 and SPP1‐CD44 interactions originating from SPP1 inflammatory TAM (Figure 4F). At the sample level, these expression‐inferred interaction scores were generally higher in NMPR than in MPR/pCR samples (Figure 4G, 4H). Together, these analyses suggest co‐occurrence of an SPP1 inflammatory TAM state with T cell dysfunction–associated transcriptional features, but they do not prove active signaling or functional macrophage–T cell suppression.

Figure 4Monocyte‐to‐TAM trajectory and SPP1‐associated macrophage–T cell interaction landscape. (A) Monocle2 trajectory constructed using monocyte, APOC1_GPNMB TAM, and SPP1 inflammatory TAM, colored by cell state annotation. (B) The same trajectory colored by pseudotime. (C) Trajectory colored by pathologic response group. (D) Violin plots showing pseudotime distributions across monocyte, APOC1_GPNMB TAM, and SPP1 inflammatory TAM. (E) Dynamic expression of representative genes along pseudotime, including APOC1, CCL2, FCN1, GPNMB, INHBA, SPP1, and VCAN. (F) Heatmap showing overall macrophage‐to‐T cell interaction strength across selected myeloid sender populations and T cell receiver populations. (G) Heatmap showing top macrophage–T cell ligand–receptor interaction pairs, highlighting major SPP1‐related interactions. (H) Heatmap showing the sample‐level landscape of representative SPP1 inflammatory TAM‐to‐T cell interactions. Colors represent feature‐wise *Z*‐scores. (I) Boxplots comparing representative SPP1 inflammatory TAM‐to‐T cell interaction scores between MPR/pCR and NMPR groups.

**Figure:**
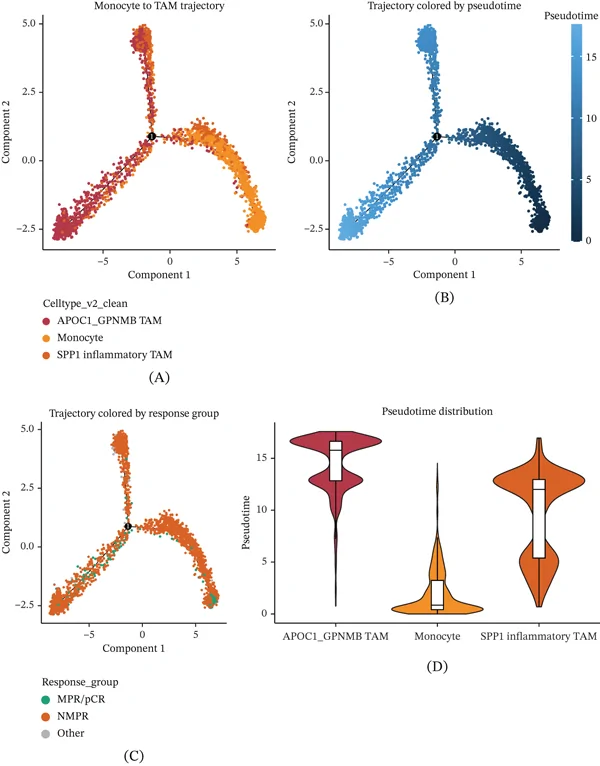
(A)

**Figure:**
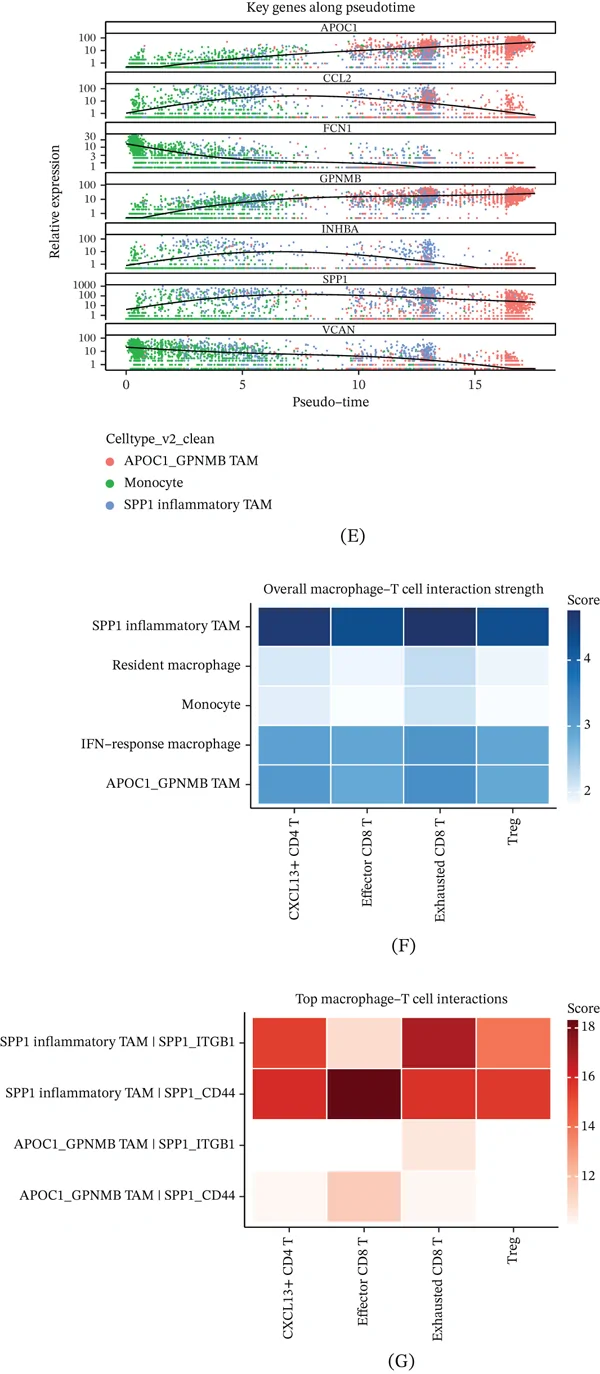
(B)

**Figure:**
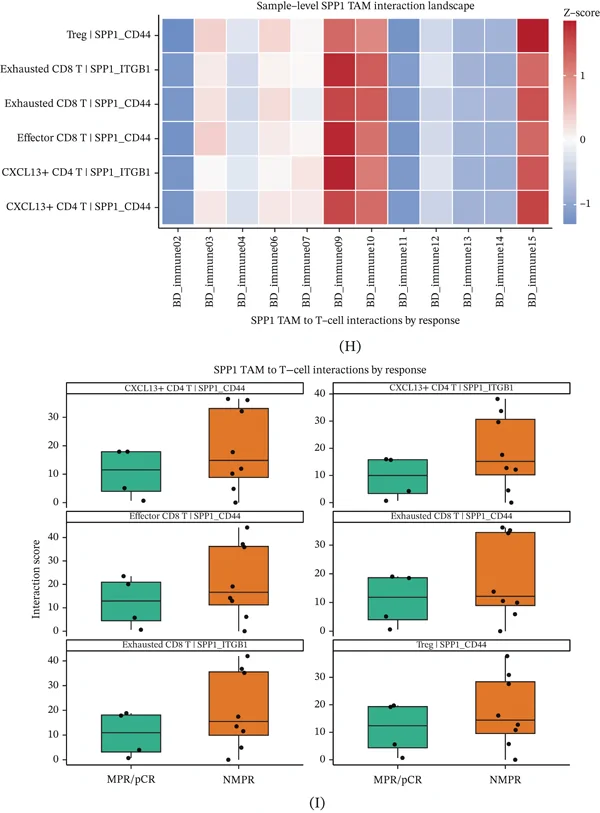
(C)

### 3.5. Projection of the NR‐TAM Program to Pretreatment Bulk RNA‐Seq

**Figure 5:**
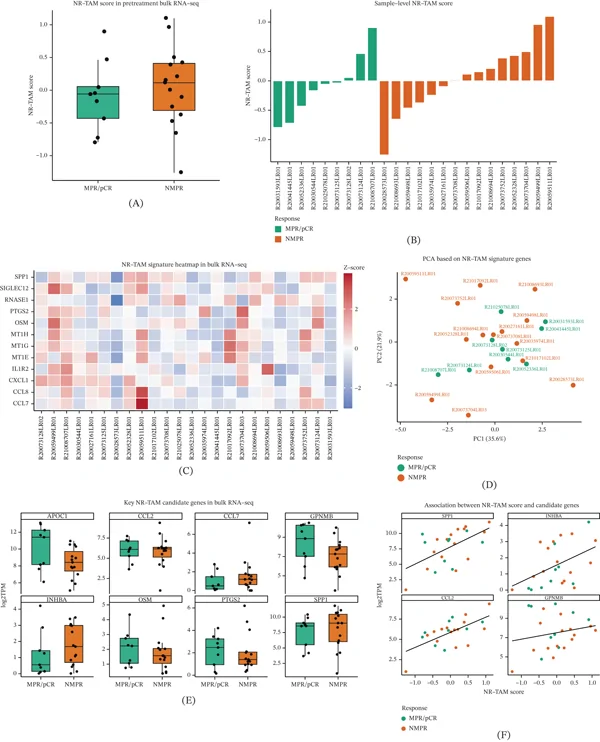
Projection of the NR‐TAM program to pretreatment bulk RNA‐seq. (A) Boxplot showing the NR‐TAM score in pretreatment bulk RNA‐seq stratified by pathologic response (MPR/pCR vs NMPR). (B) Ranked bar plot showing sample‐level NR‐TAM scores across pretreatment bulk RNA‐seq samples. Bar colors indicate pathologic response groups. (C) Heatmap showing expression of NR‐TAM signature genes across pretreatment bulk RNA‐seq samples. Colors represent gene‐wise *Z*‐scores. (D) Principal component analysis based on NR‐TAM signature genes in pretreatment bulk RNA‐seq. Points represent individual samples and are colored by pathologic response group. (E) Boxplots showing representative NR‐TAM candidate genes in pretreatment bulk RNA‐seq stratified by pathologic response. (F) Scatter plots showing associations between the NR‐TAM score and representative candidate genes. Each point represents one sample, and black lines indicate linear trends.

We next evaluated whether the adverse SPP1 inflammatory TAM‐associated program identified from posttreatment single cell data could be detected at the patient level in pretreatment bulk RNA‐seq. The resulting NR‐TAM score was overall higher in the NMPR group than in the MPR/pCR group, although overlap between groups remained evident (Figure 5A). At the individual‐sample level, most of the highest NR‐TAM scores were observed in NMPR cases, whereas several of the lowest scores were found in MPR/pCR cases, indicating interpatient heterogeneity but an overall shift toward higher scores in the poor‐response group (Figure 5B). The heat map of NR‐TAM signature genes showed coordinated but heterogeneous expression patterns across samples (Figure 5C). Genes such as SPP1, SIGLEC12, RNASE1, PTGS2, OSM, MT1H, MT1G, MT1E, IL1R2, CXCL1, CCL8, and CCL7 contributed to this transcriptional pattern, with several NMPR samples showing relatively higher expression across multiple components of the signature. PCA based on these genes further showed partial separation of response groups, with PC1 explaining 35.6% of the variance and PC2 explaining 21.9% (Figure 5D). We then examined representative candidate genes individually. In the boxplots, INHBA, CCL7, and SPP1 showed higher expression trends in the NMPR group, whereas APOC1, CCL2, GPNMB, OSM, and PTGS2 did not show the same direction of change (Figure 5E). Correlation analysis further showed that the NR‐TAM score was positively associated with SPP1, INHBA, CCL2, and GPNMB expression at the sample level (Figure 5F). Together, these results indicate that the single cell–derived NR‐TAM program can be partially recapitulated in pretreatment bulk RNA‐seq and retains a group‐level association with unfavorable pathologic response.

We further performed an exploratory ROC analysis to evaluate the discriminative capacity of the NR‐TAM score for NMPR versus MPR/pCR in pretreatment bulk RNA‐seq. The NR‐TAM score showed only modest apparent discrimination (AUC = 0.60; bootstrap 95% CI, 0.35–0.84), with substantial overlap between response groups. Using the Youden index threshold, sensitivity was 53.3%, and specificity was 77.8%. The ROC curves and summary metrics are provided in Figure S1 and Table S1. Thus, the NR‐TAM score captures a group‐level adverse response–associated signal but does not provide sufficient separation for single‐patient classification in the current cohort.

### 3.6. Clinical Relevance of INHBA and External Prognostic Evaluation in TCGA Cohorts

Given its consistent enrichment in the adverse SPP1 inflammatory TAM‐associated program, we next examined the clinical relevance of INHBA at the patient level. In pretreatment bulk RNA‐seq, INHBA expression was overall higher in the NMPR group than in the MPR/pCR group (Figure 6A). A similar pattern was observed when patients were stratified by detailed pathologic response categories, with the NMPR group remaining in the higher expression range (Figure 6B). When classified by RECIST, PR and SD cases also showed higher INHBA expression than CR cases, although intersample variability was evident (Figure 6C). We then examined INHBA expression across clinicopathologic strata. By clinical stage, INHBA expression showed an overall upward tendency from earlier to more advanced groups, with relatively higher levels observed in stage IIB samples (Figure 6D). At the individual‐sample level, the ranked bar plot further showed that several of the highest INHBA‐expressing cases belonged to the NMPR group, although overlap between response groups remained present (Figure 6E). In addition, the scatterplot showed a mild positive trend between INHBA expression and residual tumor proportion (Figure 6F). To place INHBA in the context of the TAM‐related program, we assessed its association with representative genes. INHBA showed positive trends with SPP1, CCL2, and CCL7, whereas its relationship with PTGS2 and OSM appeared relatively weak, and its trend with APOC1 was in the opposite direction (Figure 6G). These patterns support the view that INHBA is linked to part of the adverse inflammatory TAM‐associated transcriptional axis rather than uniformly tracking all macrophage‐related markers. We further examined the prognostic relevance of INHBA in external TCGA cohorts. In TCGA‐LUAD, the Kaplan–Meier curve showed a downward shift in overall survival in the high‐INHBA group, although separation between groups was modest (Figure 6H). In TCGA‐LUSC, the survival curves were more clearly separated, with the high‐INHBA group showing poorer overall survival (Figure 6I). Consistently, Cox analysis showed that the hazard estimates for INHBA were located on the risk‐increasing side in both univariate and stage‐adjusted models, with a more evident effect in LUSC than in LUAD (Figure 6J). Together, these results identify INHBA as a representative gene associated with poorer pathologic response, higher residual tumor burden, and unfavorable survival trends, supporting its prioritization for further experimental validation in subsequent work.

Figure 6Clinical relevance of INHBA in pretreatment bulk RNA‐seq and external prognostic evaluation in TCGA cohorts. (A) Boxplot showing INHBA expression in pretreatment bulk RNA‐seq stratified by pathologic response (MPR/pCR vs NMPR). (B) Boxplot showing INHBA expression across detailed pathologic response categories. (C) Boxplot showing INHBA expression across RECIST response groups (CR, PR, and SD). (D) Boxplot showing INHBA expression across clinical stages. (E) Ranked bar plot showing sample‐level INHBA expression in pretreatment bulk RNA‐seq, with bar colors indicating pathologic response groups. (F) Scatter plot showing the association between INHBA expression and residual tumor proportion. (G) Scatter plots showing associations between INHBA expression and representative TAM‐related genes, including SPP1, CCL2, CCL7, PTGS2, OSM, and APOC1. (H) Kaplan–Meier overall survival curves for INHBA high‐ and low‐expression groups in TCGA‐LUAD. (I) Kaplan–Meier overall survival curves for INHBA high‐ and low‐expression groups in TCGA‐LUSC. (J) Forest plot showing univariate and stage‐adjusted Cox regression results for INHBA in TCGA‐LUAD and TCGA‐LUSC, presented as hazard ratios with 95% confidence intervals.

**Figure:**
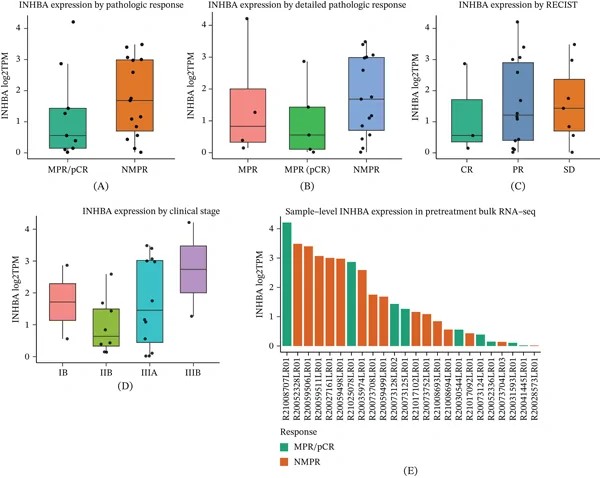
(A)

**Figure:**
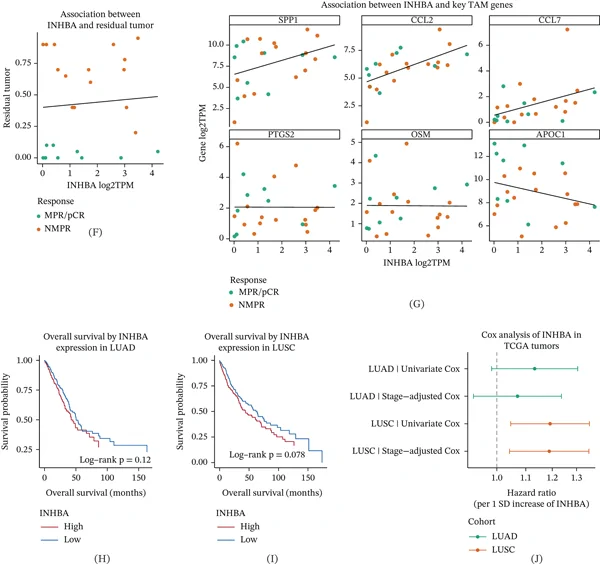
(B)

The focus on INHBA was based on concordance across several levels of analysis rather than on the assumption that it is the only relevant gene in the NR‐TAM program. Among the representative adverse response–associated genes, INHBA was enriched in SPP1 inflammatory TAM, showed higher expression trends in NMPR cases in pretreatment bulk RNA‐seq, was positively associated with residual tumor proportion and selected TAM‐related genes, showed unfavorable prognostic trends in external lung cancer cohorts, and could be tested in the available lung cancer cell line system. Other candidates such as SPP1, CCL7, CCL2, CXCL8, ADM, and GPNMB remain biologically relevant components of the program.

Exploratory performance analysis showed that INHBA expression also had modest discrimination for NMPR (AUC = 0.64; bootstrap 95% CI, 0.39–0.88). A logistic model combining NR‐TAM score and INHBA expression did not materially improve apparent discrimination (AUC = 0.64), and leave‐one‐out cross‐validation did not support robust predictive performance (AUC = 0.37). INHBA was therefore retained as a biologically prioritized candidate rather than a standalone clinical response classifier.

Because the TCGA survival trend was more evident in LUSC than in LUAD, we also examined histology distribution in the pretreatment GSE207422 bulk cohort. Squamous tumors were more frequent in the NMPR group (8/15) than in the MPR/pCR group (4/9), whereas adenocarcinoma cases were present in both response groups. This pattern suggests that the INHBA‐associated adverse signal may be influenced by histological context and should be validated separately in squamous and adenocarcinoma cohorts.

### 3.7. Experimental Validation of INHBA Expression and Function in Lung Cancer Cells

**Figure 7:**
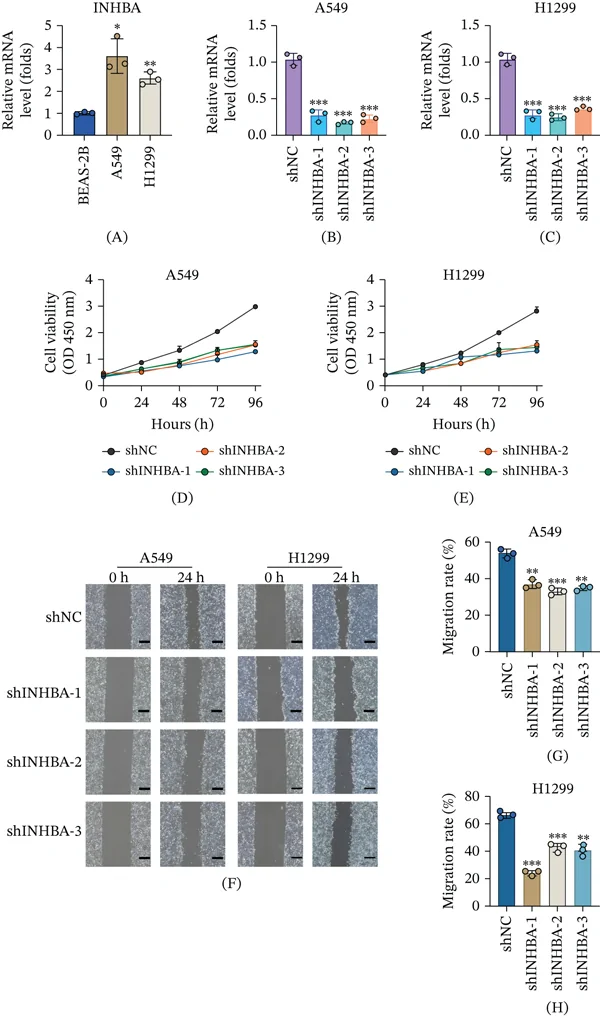
Experimental validation of INHBA expression and its functional effects in lung cancer cells. (A) qPCR analysis of INHBA expression in the normal bronchial epithelial cell line BEAS‐2B and the lung cancer cell lines A549 and H1299. Relative expression levels were normalized to GAPDH and calculated using the 2^−*ΔΔ*Ct method. (B, C) qPCR validation of INHBA knockdown efficiency in A549 (B) and H1299 (C) cells transduced with shNC or three independent INHBA‐targeting shRNAs (shINHBA‐1, shINHBA‐2, and shINHBA‐3). (D, E) CCK‐8 assays showing the proliferation of A549 (D) and H1299 (E) cells after INHBA knockdown at the indicated time points. (F) Representative wound healing images of A549 and H1299 cells transduced with shNC or INHBA‐targeting shRNAs at 0 h and 24 h. Scale bar = 100 *μ*m. (G, H) Quantification of migration rates in A549 (G) and H1299 (H) cells based on the wound healing assay. Data are presented as mean ± SD (*n* = 3). Statistical analysis was performed using one‐way ANOVA followed by Tukey′s post hoc test. In panel A, statistical significance was assessed relative to BEAS‐2B; in panels B, C, G, and H, statistical significance was assessed relative to shNC.  ^∗^P <0.05,  ^∗∗^P <0.01,  ^∗∗∗^P <0.001.

To further validate the relevance of INHBA in lung cancer, we first examined its basal expression in normal bronchial epithelial and lung cancer cell lines. qPCR showed that INHBA expression was markedly higher in A549 and H1299 cells than in BEAS‐2B cells, consistent with the transcriptomic findings indicating upregulation of INHBA in lung cancer (Figure 7A). We next established stable INHBA knockdown models in A549 and H1299 cells using three independent shRNAs. qPCR confirmed that all three shRNAs effectively reduced INHBA expression in both cell lines compared with the shNC group, indicating successful knockdown (Figure 7B,C). To assess the effect of INHBA depletion on cell growth, we performed CCK‐8 assays in A549 and H1299 cells. In both cell lines, the shNC group showed a continuous increase in cell viability over time, whereas INHBA knockdown attenuated this increase. This inhibitory effect was consistently observed across the three shRNAs, indicating that reduced INHBA expression suppressed lung cancer cell proliferation (Figure 7D,E). We then evaluated cell migration using wound healing assays. Representative images showed that wound closure at 24 h was reduced in INHBA‐silenced cells compared with shNC cells in both A549 and H1299 cell lines (Figure 7F). Quantitative analysis further confirmed that the migration rate was significantly decreased after INHBA knockdown in both cell models (Figure 7G,H). Collectively, these results support that INHBA is upregulated in lung cancer cells and contributes to the proliferative and migratory phenotypes of lung cancer cells.

## 4. Discussion

Neoadjuvant immunotherapy has reshaped the treatment landscape of resectable NSCLC, yet a substantial proportion of patients still fail to achieve a major pathologic response [45]. This clinical heterogeneity indicates that conventional biomarkers remain insufficient to fully capture the biological determinants of therapeutic response [46]. In the present study, we used an integrative single‐cell and bulk transcriptomic framework to characterize microenvironmental programs associated with pathologic response to immunotherapy in NSCLC. Our analyses identified a distinct SPP1 inflammatory TAM state enriched in samples with NMPR. This state was accompanied by increased exhausted CD8 T cell abundance, higher malignant cell hypoxia and EMT scores, and stronger SPP1‐centered macrophage–T cell interaction patterns. We further translated this single cell–derived signal to the patient level by constructing an NR‐TAM signature, and subsequently prioritized INHBA as a representative candidate associated with poorer pathologic response, higher residual tumor burden, unfavorable survival trends, and tumor‐promoting cellular phenotypes. Taken together, these findings suggest that an adverse response–associated macrophage program may be linked to an unfavorable immune microenvironment in NSCLC.

Accordingly, the results are best interpreted as associative rather than causal. The enrichment of SPP1 inflammatory TAM, exhausted CD8 T cell features, malignant cell hypoxia, and EMT programs in NMPR samples indicates co‐occurrence within an adverse response–associated ecosystem, but the current study cannot determine whether these processes are temporally ordered, mutually reinforcing, or parallel consequences of treatment pressure and aggressive tumor biology. Establishing causality will require longitudinal sampling, spatial validation, and perturbation‐based tumor‐immune models.

One important feature of this study is that the adverse microenvironmental signal was not defined simply by overall myeloid abundance, but by a more specific macrophage state. In the refined myeloid atlas, SPP1 inflammatory TAM was transcriptionally distinct from monocytes, resident macrophages, IFN response macrophages, and APOC1_GPNMB TAM. At the sample level, this population was more abundant in NMPR samples and showed coordinated positive trends with exhausted CD8 T cell proportion as well as malignant cell hypoxia and EMT programs. These observations suggest that poor pathologic response may be associated with a broader ecosystem characterized by inflammatory macrophage enrichment, T cell dysfunction, and tumor cell stress‐adaptation programs rather than by a single isolated immune feature, which is consistent with recent NSCLC‐focused work emphasizing that treatment‐relevant TAM states are more informative than bulk macrophage abundance alone [47]. Importantly, our data do not indicate that SPP1 inflammatory TAM alone is sufficient to drive treatment resistance. Rather, the results support the interpretation that this macrophage state marks an unfavorable TME in which suppressive immune remodeling and aggressive malignant cell programs tend to co‐occur [48, 49].

The trajectory analysis provides additional context for this macrophage program. Monocle‐based reconstruction suggested a continuous transition from monocyte‐like states toward terminal TAM states, with SPP1 inflammatory TAM and APOC1_GPNMB TAM occupying later branches of the trajectory. Along this transition, monocyte‐associated genes such as FCN1 and VCAN decreased, whereas macrophage‐associated and inflammatory genes including SPP1, INHBA, APOC1, GPNMB, and CCL2 increased toward later pseudotime. Although pseudotime analysis cannot establish lineage dynamics with certainty, this pattern is consistent with the idea that the adverse SPP1 inflammatory TAM state represents a more differentiated inflammatory myeloid state within the NSCLC microenvironment. Such a state may be particularly relevant in posttreatment samples, where immune remodeling has already occurred under therapeutic pressure [50, 51].

Another notable finding is the prominence of SPP1‐centered intercellular communication. Compared with other myeloid subsets, SPP1 inflammatory TAM showed stronger inferred interactions with multiple T cell populations, including exhausted CD8 T cells, effector CD8 T cells, Treg cells, and CXCL13+ CD4 T cells. Representative ligand–receptor pairs such as SPP1–ITGB1 and SPP1–CD44 were generally stronger in NMPR samples, indicating that this macrophage state may participate in a more active but potentially unfavorable immune crosstalk network. We interpret these results cautiously. The interaction scores used here reflect expression‐based inference rather than direct functional validation, and therefore should not be overinterpreted as proof of signaling dependency. Nevertheless, the convergence of abundance analysis, state scoring, pseudotime positioning, and interaction inference consistently supports the relevance of SPP1 inflammatory TAM within an adverse response–associated microenvironmental program, which is biologically plausible given recent evidence that SPP1‐positive macrophage polarity captures broader cellular programs linked to tumor progression and immune dysfunction [52].

A major strength of this work is that we did not stop at identifying a single‐cell state. Instead, we derived an NR‐TAM signature from pseudobulk analysis of SPP1 inflammatory TAM and projected it to pretreatment bulk RNA‐seq data from the same cohort. This step is important because many clinically annotated cohorts remain bulk‐based, and candidate programs defined solely at single‐cell resolution are often difficult to translate; this challenge has been specifically highlighted in recent reviews of single‐cell applications in lung cancer immunotherapy [53, 54]. In our study, the NR‐TAM signature showed higher scores in NMPR samples and captured structured transcriptional heterogeneity at the patient level. Although the separation between groups was not absolute, this is biologically expected in a clinically heterogeneous cohort and does not diminish the potential relevance of the program. Rather, it suggests that the adverse macrophage‐associated signal is one component of response variability, likely acting together with additional tumor‐intrinsic and immune‐contextual factors [55, 56].

The posttreatment origin of the single cell–derived NR‐TAM signature is an important interpretive consideration. Projecting this signature into pretreatment bulk RNA‐seq assumes that at least part of the posttreatment SPP1 inflammatory TAM program reflects a stable or recurrent adverse microenvironmental tendency that may already be detectable before therapy. However, the same program may also be amplified or reshaped by neoadjuvant treatment itself. Therefore, the pretreatment bulk analysis should be viewed as evidence of partial patient‐level recapitulation of the transcriptional program, not as proof that the SPP1 inflammatory TAM state is a purely preexisting resistance mechanism.

The exploratory predictive performance analysis also clarifies the current translational boundary of the NR‐TAM program. Although the NR‐TAM score and INHBA expression were shifted toward higher values in NMPR cases, both markers showed substantial overlap between response groups, and their apparent AUCs were modest. The combined NR‐TAM plus INHBA logistic model did not improve robust performance under leave‐one‐out cross‐validation. These results argue against using either marker as a patient‐selection tool at this stage. Rather, the NR‐TAM program should be interpreted as a biologically informative, cell state–derived signal that may complement future multiparameter models incorporating established clinical biomarkers, PD‐L1 expression, tumor mutational burden, histology, radiologic features, and other immune and stromal variables.

Practical measurement of SPP1 inflammatory TAM will also require additional development. Because this population represents a macrophage state rather than a single‐marker cell type, routine single‐marker immunohistochemistry is unlikely to be sufficient. More feasible pathology‐compatible approaches may include multiplex immunohistochemistry or immunofluorescence combining macrophage lineage markers such as CD68 or CD163 with state markers such as SPP1 and INHBA, RNA in situ hybridization panels, targeted transcriptomic assays, or spatially resolved methods in research settings. Digital pathology‐based quantification may help standardize abundance and localization metrics. Clinically validated assays and cutoffs have not yet been established.

The current results should also be viewed within a broader response landscape. SPP1 inflammatory TAM was associated with exhausted CD8 T cells, Treg‐related interactions, malignant cell hypoxia and EMT programs, and variable stromal components in the single‐cell atlas. These observations indicate that macrophages are unlikely to act in isolation. Poor pathologic response may instead reflect coordinated remodeling across myeloid, lymphoid, stromal, and tumor‐intrinsic compartments. Future models should evaluate macrophage states together with T cell functional status, fibroblast programs, malignant cell stress adaptation, and established clinicopathologic biomarkers.

Recent macrophage‐related studies in NSCLC, renal cell carcinoma, and pan‐cancer settings have developed prognostic or immunotherapy response signatures based on M1/M2 macrophage programs, TAM‐related risk genes, glycolysis‐associated macrophage programs, and pro‐inflammatory versus pro‐tumor macrophage states [47, 48, 57]. These studies support the broader relevance of macrophage heterogeneity for immune response, but many are based primarily on bulk deconvolution, prognostic endpoints, or nonneoadjuvant cohorts. The present study differs by anchoring the signature to a single‐cell‐defined SPP1 inflammatory TAM state observed in a neoadjuvant immunotherapy dataset and then projecting that state into matched pretreatment bulk RNA‐seq. At the same time, our modest AUC results underscore that biological novelty does not yet translate into clinical readiness.

Within this adverse response–associated program, INHBA emerged as a particularly informative candidate. At the single‐cell level, INHBA was enriched in SPP1 inflammatory TAM. At the bulk level, it showed higher expression in NMPR cases, a positive trend with residual tumor proportion, and coordinated relationships with several TAM‐related genes, including SPP1, CCL2, and CCL7. In external TCGA cohorts, higher INHBA expression was associated with less favorable overall survival, with a more evident trend in LUSC than in LUAD. These findings do not establish INHBA as a macrophage‐specific or NSCLC‐specific driver. However, they do support its prioritization as a representative molecule linked to an unfavorable transcriptional and clinical context, which is in line with prior evidence that INHBA is associated with aggressive behavior in NSCLC [58]. In addition, the in vitro findings that INHBA knockdown suppressed proliferative and migratory phenotypes further support its prioritization as a candidate linked to tumor‐promoting behavior [59].

The interpretation of INHBA also requires caution. In the current data, INHBA was associated with poorer pathologic response, residual tumor burden, and less favorable survival trends, particularly in LUSC. However, these observations do not establish that INHBA is an immunotherapy resistance–specific determinant. INHBA may instead reflect a broader aggressive tumor phenotype or a histology‐biased adverse microenvironmental context. Its connection to the macrophage‐associated program is supported by transcriptomic co‐enrichment and bulk‐level correlation but not by direct functional evidence that INHBA regulates macrophage state or macrophage–T cell crosstalk. The in vitro knockdown experiments support a role for INHBA in proliferation and migration, but they do not directly test immune‐checkpoint blockade sensitivity. Future experiments using tumor‐immune coculture systems, macrophage‐conditioned models, spatial validation, or immunotherapy‐relevant in vivo models will be needed to clarify whether INHBA actively contributes to immune resistance or primarily marks aggressive tumor biology.

This study has several limitations. First, the single‐cell cohort and matched pretreatment bulk cohort were relatively small, limiting statistical power and the stability of subgroup analyses. Second, the NR‐TAM score and INHBA expression showed substantial overlap between response groups, and exploratory ROC analysis demonstrated only modest discrimination; therefore, these markers are not ready for patient selection. Third, the public GSE207422 metadata did not include PD‐L1 expression, tumor mutational burden, or other routine immunotherapy biomarkers, preventing assessment of incremental predictive value beyond existing clinical markers. Fourth, the single‐cell analysis included both pretreatment and posttreatment specimens, and the NR‐TAM signature was derived largely from posttreatment SPP1 inflammatory TAM before being projected into pretreatment bulk RNA‐seq. This strategy is useful for testing partial recurrence of a single cell–derived program at the patient level, but it cannot determine whether the program is preexisting, treatment‐induced, or both. Fifth, pseudo time and macrophage–T cell communication analyses are computational inferences based on transcriptional similarity and ligand–receptor coexpression; they do not prove lineage relationships, temporal ordering, active signaling, or functional immune suppression. Sixth, the TCGA‐LUAD and TCGA‐LUSC analyses provide contextual prognostic support only, because these cohorts were not generated in neoadjuvant immunotherapy‐treated patients. Seventh, the stronger trend in LUSC than LUAD suggests potential histology dependence that requires subtype‐stratified validation. Finally, the functional assays tested tumor cell proliferation and migration after INHBA knockdown but did not directly evaluate immunotherapy sensitivity, macrophage state regulation, or tumor–immune interactions.

## 5. Conclusion

In summary, this study identifies an SPP1 inflammatory TAM program associated with unfavorable pathologic response in NSCLC treated with neoadjuvant immunotherapy and prioritizes INHBA as a candidate marker within this adverse response–associated program. These findings remain associative and exploratory, and prospective immunotherapy cohorts with matched clinical biomarkers and longitudinal tissue sampling are needed before clinical implementation or causal interpretation can be considered.

## Author Contributions

S.Z. and G.Z. contributed equally to this work. S.Z. conducted the computational analyses and prepared the initial draft. G.Z. contributed to data curation and analytical interpretation. W.W. and H.L. assisted with data organization and manuscript revision. H.C. designed the study, supervised the project, and finalized the manuscript. S.Z. and G.Z. have contributed to the work equally and should be regarded as cofirst authors.

## Funding

No funding was received for this manuscript.

## Disclosure

All authors read and approved the final version.

## Ethics Statement

This study was based exclusively on publicly available datasets and did not involve direct collection of human or animal samples by the authors. Therefore, ethical approval and informed consent were not required for this study.

## Conflicts of Interest

The authors declare no conflicts of interest.

## Supporting Information

Additional supporting information can be found online in the Supporting Information section.

## Supporting information


**Supporting Information 1** Figure S1: The exploratory ROC curves assessing the discrimination of NMPR by the NR‐TAM score, INHBA expression, and their combined logistic model in the pretreatment bulk RNA‐seq cohort.

**Supporting Information 2** Table S1: The corresponding predictive performance statistics, group descriptives, histology counts, NR‐TAM signature genes, and sample‐level scores (Figure S1 and Table S1).

## Data Availability

The datasets analyzed in this study are publicly available in GEO under accession number GSE207422 and in TCGA. Data generated from the in vitro experiments are included in the article, and additional information is available from the corresponding author upon reasonable request.
