# Supplementary material for: Single‐Cell and Bulk Transcriptomic Integration Reveals an Adverse SPP1 Inflammatory TAM State and INHBA as a Candidate Biomarker in Non‐Small Cell Lung Cancer Immunotherapy
Source: Int J Genomics. 2026 Aug 26;2026:2321522. doi: 10.1155/ijog/2321522 (PMC13508024; doi:10.1155/ijog/2321522)
Supplement: Supplementary file 1 [file IJOG-2026-2321522-s002.pdf]

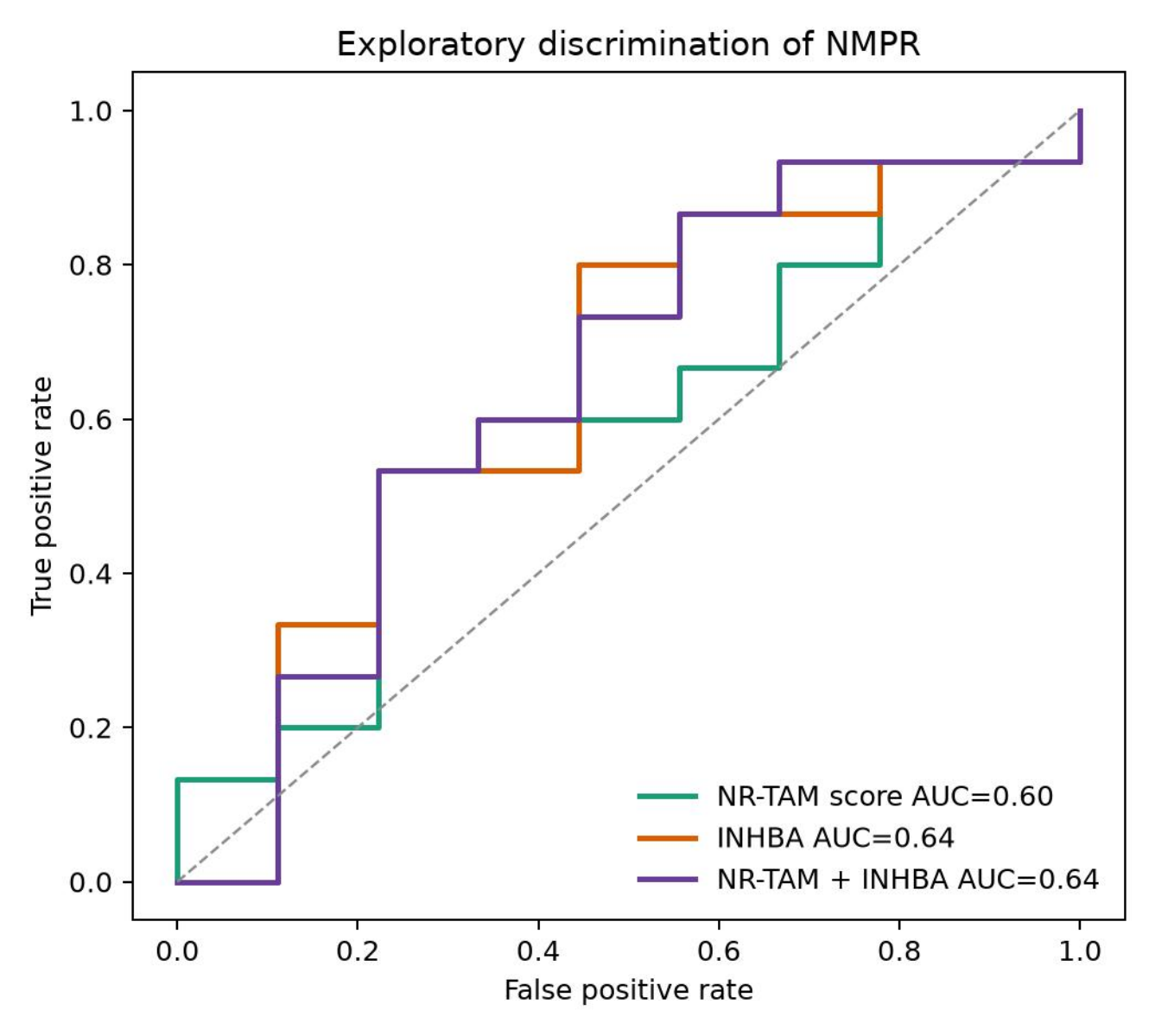

Exploratory discrimination of NMPR
True positive rate
False positive rate
0.0
0.2
0.4
0.6
0.8
1.0
NR-TAM score AUC=0.60
INHBA AUC=0.64
NR-TAM + INHBA AUC=0.64
